# High-Sensitivity Optical Fiber-Based Glucose Sensor Using Helical Intermediate-Period Fiber Grating

**DOI:** 10.3390/s22186824

**Published:** 2022-09-09

**Authors:** Junlan Zhong, Shen Liu, Tao Zou, Wenqi Yan, Peijing Chen, Bonan Liu, Zhongyuan Sun, Yiping Wang

**Affiliations:** 1 Key Laboratory of Optoelectronic Devices and Systems of Ministry of Education/Guangdong Province, College of Physics and Optoelectronic Engineering, Shenzhen University, Shenzhen 518060, China; 2 Shenzhen Key Laboratory of Photonic Devices and Sensing Systems for Internet of Tings, Guangdong and Hong Kong Joint Research Centre for Optical Fibre Sensors, Shenzhen University, Shenzhen 518060, China

**Keywords:** helical intermediate-period fiber grating, optical fiber sensor, glucose detection

## Abstract

An all-fiber glucose sensor is proposed and demonstrated based on a helical intermediate-period fiber grating (HIPFG) produced by using a hydrogen/oxygen flame heating method. The HIPFG, with a grating length of 1.7 cm and a period of 35 μm, presents four sets of double dips with low insertion losses and strong coupling strengths in the transmission spectrum. The HIPFG possesses an averaged refractive index (RI) sensitivity of 213.6 nm/RIU nm/RIU in the RI range of 1.33–1.36 and a highest RI sensitivity of 472 nm/RIU at RI of 1.395. In addition, the HIPFG is demonstrated with a low-temperature sensitivity of 3.67 pm/°C, which promises a self-temperature compensation in glucose detection. In the glucose-sensing test, the HIPFG sensor manifests a detection sensitivity of 0.026 nm/(mg/mL) and a limit of detection (LOD) of 1 mg/mL. Moreover, the HIPFG sensor exhibits good stability in 2 h, indicating its capacity for long-time detection. The properties of easy fabrication, high flexibility, insensitivity to temperature, and good stability of the proposed HIPFG endow it with a promising potential for long-term and compact biosensors.

## 1. Introduction

Monitoring glucose concentrations is critical for human health, and glucose sensors have gotten much interest in medical diagnostics and treatment. Diabetes is caused by persistent hyperglycemia (blood glucose levels exceeding 2.3 mg/mL), leading to significant health consequences such as heart disease, stroke, and kidney failure. Hypoglycemia (blood sugar levels below 0.65 mg/mL) can cause dizziness, difficulty concentrating, and even shock. Several approaches have been developed for glucose detection, including electrode methods, chemiluminescence, and electrochemical methods, all of which are usually time-consuming and complex [1,2,3,4]. Therefore, easy-to-handle glucose level detection with excellent sensitivity is a critical diagnostic technique for glucose concentration monitoring.

In recent years, optical fiber sensors (OFSs) have been intensively investigated as an appropriate platform for glucose detection, with strong biocompatibility, simplicity of integration, and low cost, to address the limitations of conventional biosensors [5,6,7,8,9,10,11,12,13]. Most OFSs employ the change in transmitted or reflected light caused by RI changes in the target sample as their sensing principle. In 2009, Binu et al. presented a glucose detecting system based on polymethyl methacrylate (PMMA) fiber and applied it to glucose measurement with concentrations in the range of 0–25% [5]. Deep et al. described a glucose sensor based on a long-period fiber grating (LPFG) with a glucose sensitivity of 0.806 nm/(mg/mL) at a glucose concentration in the range of 10–300 mg/dL and temperature sensitivity of 300 pm/°C [6]. In 2014, Luo et al. proposed a tilted fiber grating (TFG)-based sensor for glucose measurement with a sensitivity of 0.298 nm/(mg/mL) in the low concentration range of 0.0~3.0 mg/mL. The TFG-based sensor has a relatively lower temperature sensitivity (~5.3 pm/°C) than LPFG-based sensors [12]. Some sensitive compounds were immobilized on the fiber surface to increase sensitivity. For example, Jiang et al. deposited GO on the surface of the TFG in 2018, and their experimental results indicated that the sensor’s sensitivity for detecting glucose concentration was around 1.33 nm/mg/mL in the range of 0–8 mM [13]. OFSs based on surface-plasmon resonance (SPR), a Mach–Zehnder interferometer (MZI), photonic crystal fiber (PCF), and whispering gallery mode (WGM) resonances are also commonly utilized in glucose detection [14,15,16,17]. Abdulhalim demonstrated a blood glucose biosensor utilizing an SPR with a sensitivity of 0.14 nm/(mg/dL) with a glucose concentration of 0–200 mg/dL [14]. Biosensors based on a multimode microfiber with refractive index (RI) sensitivity of 2180 nm/RIU and glucose-sensing sensitivity of 1.74 nm/(mg/mL) were described by Li et al. in 2018 [15]. More recently, in 2019, an SPR-based sensor, fabricated using D-shaped PCF, was reported by Lidiya et al., and the results showed a sensitivity of 0.83 nm/(mg/mL) in the glucose concentration range of 0–100 mg/mL [16]. Brice et al. proposed a glucose sensor based on WGM by hybridizing the WGMRs with gold nanoparticles [17].

A glucose sensor based on a helix intermediate-period fiber grating (HIPFG) with a high RI and low-temperature sensitivity is demonstrated first in this paper. The HIPFG is defined as an intermediate-period fiber grating with a helical structure and intermediate period smaller than 100 μm, which possesses the advantages of high sensitivity, small insertion loss resistance, good mechanical strength, high stability, and insensitivity to temperature and torsion [18,19]. This work is a follow-up work of our previous work reported by Zou et al. and Zhong et al. [19,20]. By comparing with the E-HIPFG, a HIPFG inscribed in elliptical core fiber, the HIPFG written in single-mode fiber has superior fabrication efficiency and reproducibility [20]. The HIPFG inscribed in single-mode fiber (SMF) by a hydrogen/oxygen flame heating system has a highest RI sensitivity of 472 nm/RIU at a dip around 1530 nm. The temperature sensitivity is 3.67 pm/°C, confirming the insensitivity of the HIPFG to temperature interference. Twenty-six glucose solutions, with concentrations ranging from 0.02 mg/mL to 200 mg/mL, were used to identify the glucose properties of the proposed HIPFG. The results reveal that the HIPFG gives a glucose detection sensitivity of 0.026 nm/(mg/mL) and a limit of detection (LOD) of 1 mg/mL. The exceptional stability of the HIPFG sensor is also proved for 2 h with an average error of 0.001 nm. To the best of our knowledge, this is the first paper to realize glucose detection by using HIPFG.

## 2. Materials and Methods

**Figure 1:**
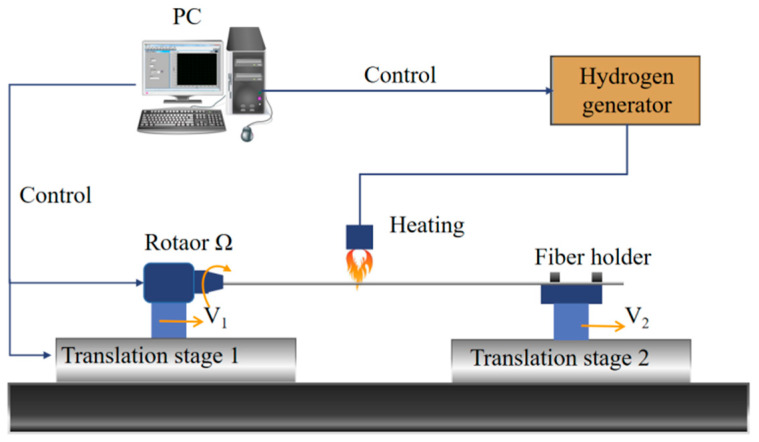
Schematic of grating fabrication configuration using a hydrogen/oxygen flame.

To fabricate the HIPFG for our experiment, we used high-efficiency helical grating manufacturing equipment that included a high-precision rotator, two translation stages, a hydrogen generator, and a LabVIEW program control portion, as shown in Figure 1. The hydrogen/oxygen flame heating system for processing helical fiber gratings has several benefits, including high reproductivity, high efficiency, and good grating quality. Those qualities have been demonstrated in our previous publications [19,20,21,22,23,24]. The HIPFG was written with a rotation rate Ω of 2057 rpm, a velocity of 1.10 mm/s for the left translational stage V_1_, and a velocity of 1.20 mm/s for the right translational stage V_2_ when the SMF (Corning, SMF-28) was melting by hydrogen/oxygen flame heating.

**Figure 2:**
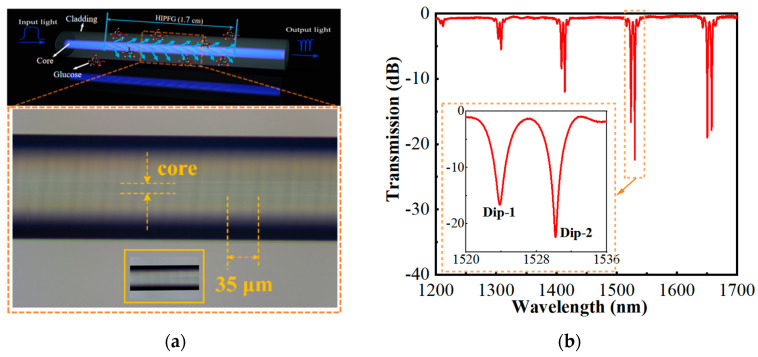

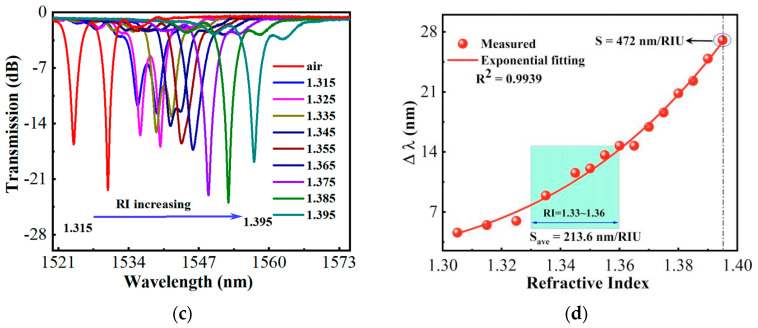
(**a**) Schematic configuration of the HIPFG sensor and the real structure of the fabricated HIPFG observed by the optical microscope; (**b**) transmission spectrum of the fabricated HIPFG measured in the air; (**c**) transmission spectrum of the fabricated HIPFG measured in different RI-matching liquid; (**d**) relationship between the RI and the wavelength shift of Dip−1.

The schematic configuration of the HIPFG sensor and the real structure of the fabricated HIPFG are shown in Figure 2a. The upper figure of Figure 2a represents the 3D diagram of the proposed HIPFG sensor, while the lower figure depicts the actual structure of the HIPFG obtained by an optical microscope. The periodic RI perturbations are effectively produced in the core of SMF without mechanical deformation on the HIPFG surface, as illustrated in the picture. The inset figure of lower Figure 2a shows the grating structure of the fabricated HIPFG. The grating period was calculated to be 35 μm by the formula Λ = 60 V_2_/Ω and the grating length was cut to be 17 mm.

In the measurement system, one end of the HIPFG was joined by an amplified spontaneous light source (ASE, NKT Photonics) to emit light into the HIPFG. Another end was connected to an optical spectrum analyzer (OSA, Yokogawa, AQ6370C) for monitoring the transmission spectra of the HIPGH. Figure 2b presents the transmission spectrum of the HIPFG in air measured with a wavelength range of 1200–1700 nm and a resolution of 0.05 nm. A series of dual dips emerge in the spectrum because the wave vectors in those dip wavelengths fulfill the phase match condition (PMC), while the dual dips are caused by the TE and TM polarizations of the fundamental core mode, which couple into forward-propagating TE and TM cladding modes, respectively [19,25,26]. Among those double-dip sets, the dips around 1530 nm have the highest coupling strength with a coupling strength of 16.67 dB for the peak at 1523.94 nm (Dip−1) and 22.40 dB for the peak at 1530.23 nm (Dip−2), as shown in the inset figure of Figure 2b.

To study the sensing performance, the RI sensing test was carried out. A succession of typical RI-matching solutions was used to submerge the HIPFG (provided by Cargille Labs). To obtain a stable condition, the transmission spectra were recorded a few minutes after the immersion. The RI-matching liquid on the surface of the HIPFG was cleaned with alcohol after each measurement until the spectrum was restored to its original condition. Figure 2c shows the HIPFG transmission spectra in air and different RI-matching liquids with a RI ranging from 1.305 to 1.395. The peak moves toward a longer wavelength as the RI rises. In particular, the separated peaks of HIPFGs merge when the RI is above 1.355. This is because the eigenvalue equation of the TM cladding mode is influenced by the surrounding medium RI and the index difference between the cladding and the surrounding medium. Therefore, the index gap between these TM and TE cladding modes decreases as the surrounding refractive index rises [26]. The connection between the wavelength shifts (Δλ = λ_sample_ – λ_air_) in various RI-matching liquids is determined by comparing the wavelength of Dip-1 in air, as illustrated in Figure 2d. The sensor’s RI sensitivity appears to increase nonlinearly with the increase of the surrounding RI. This result is consistent with the results of formal reports, which demonstrated that the shift of resonant wavelength exhibits a nonlinear function with increasing surrounding RI [27,28]. The red line is the exponential fitting line of the experimental data, with an R^2^ of 0.9939. When the surrounding RI is 1.3, the HIPFG has a low RI sensitivity of 93 nm/RIU. The average sensitivity (S_ave_) of the HIPFG in the RI range of 1.33–1.36 is calculated to be 213.6 nm/RIU. The RI of glucose solution is nearly located in 1.33–1.36; therefore, the average sensitivity in this range is vital to evaluate the sensing performance. As the surrounding RI increases to 1.395, the HIPFG shows the highest sensitivity of 472 nm/RIU.

**Figure 3:**
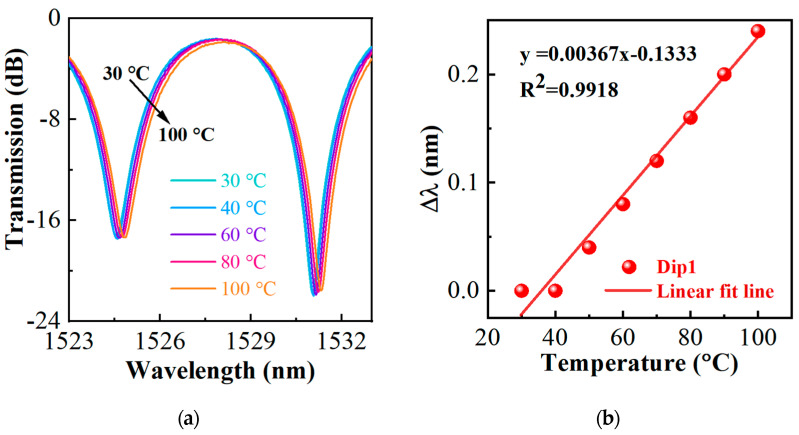
(**a**) Transmission spectra of the HIPFG with increasing temperatures. (**b**) Relationship between the temperature and the wavelength shift of Dip−1.

By placing the HIPFG sensor in a column oven with a temperature resolution of 0.1 °C, the temperature response of the sensor is studied. Figure 3 exhibits the transmission spectra of the HIPFG response to the temperature from 30 to 100 °C with an interval of 10 °C. As the temperature increases, the wavelengths of the double dips slightly shift to the long-wavelength side. Figure 3b indicates the wavelength shift of Dip−1 in response to the temperature change. The red line is the linear fitting line of Dip−1, for which R^2^ equals 0.9918, and the temperature sensitivity of the HIPFG is calculated to be 3.67 pm/°C. This result is lower than that of the previously reported optical fiber sensors [19]. Therefore, the effect of temperature cross-sensitivity caused by this sensor is significantly reduced.

## 3. Glucose-Sensing Properties of the HIPFG

**Figure 4:**
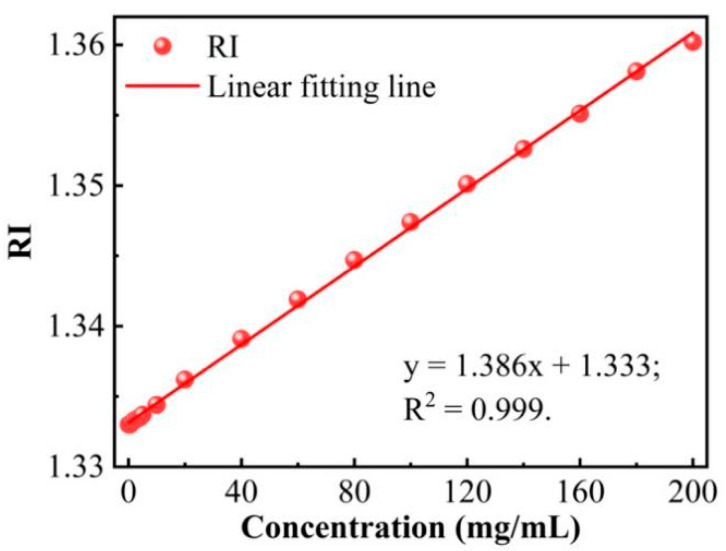
Relationship between the glucose concentration and the RI.

D(+)-glucose was bought from Sigma-Aldrich International GmbH (China). In our experiment, deionized (DI) water and 26 glucose solutions with concentrations ranging from 0.02 to 200 mg/mL were used for glucose sensitivity measurement. The samples were prepared by diluting different masses of glucose powder into 10 mL of DI water. The Ris of each sample were measured by a Table Digital Refractometer (TDR095). The corresponding RI value range of those glucose samples is 1.3330–1.3602 and the relationship between the glucose concentration and the RI is illustrated in Figure 4. The HIPFG was packed in a glass dish before being detected in the experiment. By syringe, glucose solutions of various concentrations were delivered into the glass dish. The previously used glucose solution was removed after each measurement. The glass dish and HIPFG were thoroughly cleaned with DI water and acetone. The measurement was carried out in a range of 1535–1555 nm and with a resolution of 0.02 nm.

The HIPFG couples the light from the fundamental core mode to the forward-propagating cladding modes leading to the distinct attenuation dips in the transmission spectrum at resonant wavelengths satisfying the following equation [29]:

(1) $$\lambda_{i}=(n_{core}-n_{cladding}^{i})\Lambda ,$$

where $n_{core}$ and $n_{cladding}^{i}$ are the effective refractive index of the fundamental core mode and the *i*th order cladding mode, respectively, and *λ_i_* is the *i*th order resonance wavelength, and Λ is the grating period. Equation (1) demonstrates that the resonant wavelength *λ_i_* is mainly influenced by the evanescent field of the cladding mode, in other words, the change of the surrounding RI. Therefore, by monitoring the variations of the resonant wavelength in the transmission spectroscopy of the HILPG, the RI changes of the surrounding medium linked to the target sample can be observed.

**Figure 5:**
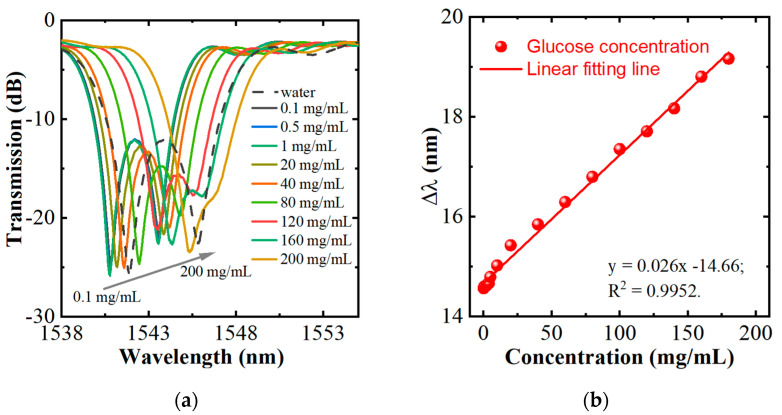
(**a**) Transmission spectra of water and glucose solutions measured by the HIPFG; (**b**) relationship between the glucose concentration and the wavelength shift of Dip−1.

Figure 5a shows the transmission spectra in 1538–1553 nm of HIPFG in water and glucose solutions. Figure 5a shows the transmission spectra of water and glucose solutions measured by the HIPFG. The spectrum of water (The dashed gray line) shows two dips at 1540.73 nm and 1543.51 nm with losses of −25.67 dB and −22.59 nm, respectively. The transmission spectra of glucose solutions show a similar spectral shape to that of water but with dips at longer wavelengths and lower losses. As the glucose concentration increases, the dips shift to longer wavelengths, and the wavelength difference between the two dips becomes blurred. This phenomenon can be attributed to the increase in glucose concentration increasing the RI of the surrounding medium [30], consequently leading the mode indexes of the polarization states to decrease. Figure 5b shows the sensor response of the concentrations of glucose solutions and the dip wavelength of Dip-1. The glucose sensitivity (S) is 0.026 nm/(mg/mL). The LOD can be calculated by the equation of LOD = δ*λ*/S, where δ*λ* is the wavelength resolution of OSA. Because of the resolution of 0.02 nm of the OSA, the LOD of the HIPFG sensor is calculated to be 1 mg/mL.

**Figure 6:**
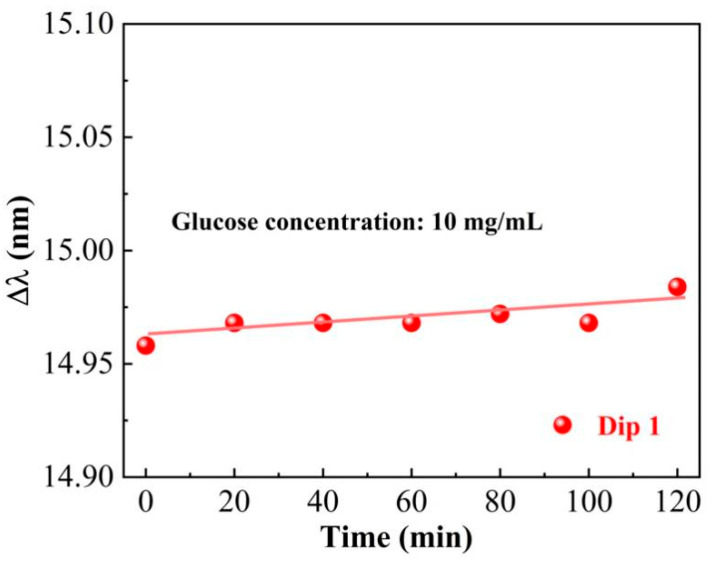
Sensing stability of the HIPFG with the wavelength shift of Dip-1.

To test the stability of the HIPFG sensor, it was dipped in a glucose solution with a concentration of 10 mg/mL, and the transmission spectrum was measured every 20 min for two hours. The wavelength shifts about 14.96 nm when the HIPFG was merging in a glucose solution, with a root mean squared error (calculated by $RMSE=\sqrt{\frac{\sum_{i=1}^{n}{\left(x_{i}-\bar{x}\right)}^{2}}{n}}$) of 0.007 nm in two hours, as shown in Figure 6. This result confirms the HIPFG sensor’s outstanding stability, which is vital for long-term monitoring.

Table 1 illustrates the comparison of glucose-sensing performances of optical fiber gratings. Most grating sensors reported in previous articles are combined with glucose-sensitive materials, such as GO (graphene oxide) and GOD (glucose oxidase). However, the sensing performances are affected by the combination of the sensitive material and the optical fiber sensor, making it more difficult to build durable sensors. The suggested HIPFG sensor offers a novel approach to glucose monitoring avoiding material combination. Its stability has been demonstrated above, which ensures its durability for long-term application. Furthermore, because of its low-temperature sensitivity of 3.67 pm/°C, which is negligible compared to the glucose-induced wavelength shift, HIPFG has unique stability in a temperature-changing environment.

## 4. Conclusions

In this work, a biosensor for glucose detection based on E-HIPFG was fabricated by a hydrogen/oxygen flame heating system, and the optical working characteristics of the sensor were examined. At the peak around 1530 nm, the RI sensitivity increases nonlinearly with the surrounding RI, and the highest RI sensitivity of 472 nm/RUI is archived in the RI range of 1.305–1.395. Furthermore, the experimented HIPFG presents low-temperature sensitivities of 3.67 pm/°C with the temperature ranging from 30 °C to 100 °C. Various concentrations of the glucose solution ranging from 0.02 mg/mL to 200 mg/mL, with a corresponding RI range of 1.3330–1.3602, were measured to identify the concentration sensitivity of the proposed HIPFG. The results significantly demonstrate the glucose sensor sensitivity of 0.02 nm/(mg/mL) and a LOD of 1 mg/mL. In addition, the HIPFG sensor shows good stability in 2 h and can be employed for long-time detection. The proposed sensor can be developed as a powerful sensing platform for drug discovery, clinical diagnostics, and food safety applications with the benefits of probability, easy fabrication, strong anti-interference capability, and considerable stability.

## Figures and Tables

**Table 1:** Comparison of glucose-sensing performances of optical fiber gratings with this work.

Sensor Type	External Material	SRI Sensitivity	Sensing Range	Glucose Sensitivity	Temperature Sensitivity	Ref.
TFG	GO ^1^ and GOD ^2^	128 nm/RIU	0–8 mM	1.33 nm/(mg/mL)	-	[13]
LPG	GOD	-	0–1.2 mg/mL	0.77 nm/(mg/mL)	-	[10]
S-shape LPG	GOD	-	0–1%	0.64 dB/(mg/mL)	-	[11]
HIPFG	Not required	472 nm/RIU	0–200 mg/mL	0.026 nm/(mg/mL)	3.67 pm/°C	This work

^1^ Graphene oxide, ^2^ glucose oxidase.

## Data Availability

Not applicable.

## References

[1] Yan K., Zhang D., Wu D., Wei H., Lu G.M. (2014). Design of a breath analysis system for diabetes screening and blood glucose level prediction. IEEE Trans. Biomed. Eng..

[2] Chen C., Xie Q.J., Yang D.W., Xiao H.L., Fu Y.C., Tan Y.M., Yao S.Z. (2013). Recent advances in electrochemical glucose biosensors: A review. Rsc Adv..

[3] Yu J.H., Ge L., Huang J.D., Wang S.M., Ge S.G. (2011). Microfluidic paper-based chemiluminescence biosensor for simultaneous determination of glucose and uric acid. Lab Chip.

[4] Liu Q.Y., Li H., Zhao Q., Zhu R., Yang Y., Jia Q., Bian B., Zhuo L. (2014). Glucose-sensitive colorimetric sensor based on peroxidase mimics activity of porphyrin-Fe_3_O_4_ nanocomposites. Mater. Sci. Eng. C.

[5] Binu S., Pillai V.P.M., Pradeepkumar V., Padhy B.B., Joseph C.S., Chandrasekaran N. (2009). Fibre optic glucose sensor. Mater. Sci. Eng. C.

[6] Deep A., Tiwari U., Kumar P., Mishra V., Jain S.C., Singh N., Kapur P., Bharadwaj L.M. (2012). Immobilization of enzyme on long period grating fibers for sensitive glucose detection. Biosens. Bioelectron..

[7] Badmos A.A., Sun Q.Z., Sun Z.Y., Zhang J.X., Yan Z.J., Lutsyk P., Rozhin A., Zhang L. (2007). Enzyme-functionalized thin-cladding long-period fiber grating in transition mode at dispersion turning point for sugar-level and glucose detection. J. Biomed. Opt..

[8] Yang R.Z., Dong W.F., Meng X., Zhang X.L., Sun Y.L., Hao Y.W., Guo J.C., Zhang W.Y., Yu Y.S., Song J.F. (2012). Nanoporous TiO_2_/polyion thin-film-coated long-period grating sensors for the direct measurement of low-molecular-weight analytes. Langmuir.

[9] Novais S., Ferreira C.I.A., Ferreira M.S., Pinto A.J.L. (2018). Optical fiber tip sensor for the measurement of glucose aqueous solutions. IEEE Photonics J..

[10] Xu B., Huang J., Ding L.Y., Cai J. (2020). Graphene oxide-functionalized long period fiber grating for ultrafast label-free glucose biosensor. Mater. Sci. Eng. C.

[11] Wu C.W. (2020). S-shaped long period fiber grating glucose concentration biosensor based on immobilized glucose oxidase. Optik.

[12] Luo B.B., Yan Z.J., Sun Z.Y., Li J.F., Zhang L. (2014). Novel glucose sensor based on enzyme-immobilized 81 tilted fiber grating. Opt. Express.

[13] Jiang B.Q., Zhou K.M., Wang C.L., Sun Q.Z., Yin G.L., Tai Z.J., Wilsone K., Zhao J.L., Zhang L. (2018). Label-free glucose biosensor based on enzymatic graphene oxide-functionalized tilted fiber grating. Sens. Actuator B Chem..

[14] Srivastava S.K., Abdulhalim I. (2015). Spectral interrogation based SPR sensor for blood glucose detection with improved sensitivity and stability. Biosens. Bioelectron..

[15] Li Y.P., Ma H., Gan L., Liu Q., Yan Z.J., Liu D.M., Sun Q.Z. (2018). Immobilized optical fiber microprobe for selective and high sensitive glucose detection. Sens. Actuators B.

[16] Lidiya A.E., Raja R.V.J., Ngo M.Q., Vigneswaran D. (2019). Detecting hemoglobin content blood glucose using surface plasmon resonance in D-shaped photonic crystal fiber. Opt. Fiber Technol..

[17] Brice I., Grundsteins K., Atvars A., Alnis J., Viter R., Ramanavicius A. (2020). Whispering gallery mode resonator and glucose oxidase based glucose biosensor. Sens. Actuators B Chem..

[18] Kopp V.I., Churikov V.M., Singer J., Chao N., Neugroschl D., Genack A.Z. (2004). Chiral fiber gratings. Science.

[19] Zou T., Zhong J.L., Liu S., Zhu G.X., Zhao Y.Y., Luo J.X., Lu S.Z., Zhang Q., He J., Bai Z.Y. (2021). Helical Intermediate-period Fiber Grating for Refractive Index Measurements with Low-Sensitive Temperature and Torsion Response. J. Light. Technol..

[20] Zhong J.L., Liu S., Zou T., Yan W.Q., Zhou M., Liu B.N., Rao X., Wang Y., Sun Z.Y., Wang A.P. (2022). All Fiber-Optic Immunosensors Based on Elliptical Core Helical Intermediate-Period Fiber Grating with Low-Sensitivity to Environmental Disturbances. Biosensors.

[21] Liu S., Zhang Y., Fu C.L., Bai Z.Y., Li Z.L., Liao C.R., Wang Y., He J., Liu Y., Wang Y.P. (2018). Temperature insensitivity polarization-controlled orbital angular momentum mode converter based on an LPFG induced in four-mode fiber. Sensors.

[22] Zhao Y.Y., Liu S., Luo J.X., Chen Y.P., Fu C.L., Xiong C., Wang Y., Jing S.Y., Bai Z.Y., Liao C.R. (2020). Torsion, Refractive Index, and Temperature Sensors Based on An Improved Helical Long Period Fiber Grating. J. Lightwave Technol..

[23] Fu C.L., Wang Y.P., Liu S., Bai Z.Y., Tang J., Shao L.P., Liu X.Y. (2019). Transverse-load, strain, temperature, and torsion sensors based on a helical photonic crystal fiber. Opt. Lett..

[24] Liu S., Yan W.Q., Zhong J.L., Zou T., Zhou M., Chen P.J., Xiao H., Liu B.N., Bai Z.Y., Wang Y.P. (2022). Compact breath monitoring based on helical intermediate-period fiber grating. Sens. Actuator B Chem..

[25] Yan Z.J., Wang H.S., Wang C.L., Sun Z.Y., Yin G.L., Zhou K.M., Wang Y.S., Zhao W., Zhang L. (2016). Theoretical and experimental analysis of excessively tilted fiber gratings. Opt. Express.

[26] Feng D.Y., Li Z.H., Zheng H.R., Jiang B.Q., Albert J., Zhao J.L. (2020). Strong cladding mode excitation in ultrathin fiber inscribed Bragg grating with ultraviolet photosensitivity. Opt. Express.

[27] Xuewen S., Lin Z., Bennion I. (2002). Sensitivity characteristics of long-period fiber gratings. J. Lightwave Technol..

[28] Kurmoo Y., Hook A.L., Harvey D., Dubern J.F., Williams P., Morgan S.P., Korposh S., Alexander M.R. (2020). Real time monitoring of biofilm formation on coated medical devices for the reduction and interception of bacterial infections. Biomater. Sci..

[29] Gong P.Q., Li X.G., Zhou X., Zhang Y.N., Chen N., Wang S.K., Zhang S.Q., Zhao Y. (2021). Optical fiber sensors for glucose concentration measurement: A review. Opt. Laser Technol..

[30] Yasin M., Irawati N., Harun S.W., Ahmad F., Khasana M. (2019). Sodium nitrate (NaNO_3_) sensor based on graphene coated microfiber. Measurement.

